# Editorial: Transcriptional regulation in cardiovascular diseases

**DOI:** 10.3389/fcvm.2024.1360765

**Published:** 2024-01-17

**Authors:** Yali Nie, Chao Song, Huifang Tang

**Affiliations:** ^1^ Hunan Provincial Key Laboratory of Multi-Omics and Artificial Intelligence of Cardiovascular Diseases, University of South China, Hengyang, Hunan, China; ^2^ Department of Cardiology, The First Affiliated Hospital, Hengyang Medical School, University of South China, Hengyang, Hunan, China; ^3^ Department of Cardiology, The First Affiliated Hospital, Institute of Cardiovascular Disease, Hengyang Medical School, University of South China, Hengyang, Hunan, China; ^4^ Clinical Research Center for Myocardial Injury in Hunan Province, Hengyang, Hunan, China; ^5^ Cardiovascular Lab of Big Data and Imaging Artificial Intelligence, The First Affiliated Hospital, Hengyang Medical School, University of South China, Hengyang, Hunan, China

**Keywords:** transcriptional regulation, cardiovascular diseases, transcription factors, bioinformatics analysis techniques, prospect

**Editorial on the Research Topic** Transcriptional regulation in cardiovascular diseases

## Introduction

Cardiovascular diseases are the most dangerous diseases, which can cause dysfunction of the human circulatory system, and eventually lead to an increased risk of sudden cardiac death (1, 2). Emerging evidence revealed that transcriptional mis-regulation induced abnormal gene expression is one of the major causes of multiple cardiovascular diseases (3–5). Transcriptional regulation is a complex process that governs gene expression by facilitating communication between DNA regulatory elements and transcriptional regulators. With the development of sequencing techniques and release of abundant cardiovascular multi-omics data, identification of causal transcriptional regulators or cis-regulatory elements from omics perspectives is the effective method to unveil the pathological molecular mechanisms and to develop novel pharmacological strategies for cardiovascular diseases.

Recently, several studies have highlighted the biological function of several transcription regulators in the development of cardiovascular disease, such as Tbx1 inhibits the autoimmune response within the area of myocardial infarction, IRX2 induces levels of myocardial fibrosis in dilated cardiomyopathy, and BACH1 promotes the dedifferentiation phenotype of vascular smooth muscle cells (6–8). Mechanistically, transcriptional regulation, which transmits genetic information from DNA into RNA, is the most critical step in the nucleus of eukaryotic cells. The process of transcriptional regulation occurs in proximal promoter regions or distal enhancer regions and is manipulated by multiple transcription factors, chromatin remodeling complexes, and transcriptional cofactors (9, 10). Moreover, a variety of transcription factors-enhancer-promoter transcriptional regulatory circuits are formed under precise regulation to promote the transcriptional expression of various genes. Any abnormality in the transcriptional regulatory process can affect the occurrence of cardiovascular disease. Therefore, further exploration of various mechanisms of transcriptional regulation involved in the occurrence of cardiovascular diseases is helpful to improve the understanding of the pathogenesis of cardiovascular diseases. Thus, this has become the main direction of current thematic research. A total of 4 articles (3 research articles, 1 review) were published in this research topic, and the unique contributions of each article are summarized below.

### Transcription regulation between macrophages and cardiovascular disease

The anti-inflammatory and pro-inflammatory properties of macrophages make them not only have heart repair effects, but also inflammatory damage effects in the development of cardiovascular diseases (11). Existing studies have demonstrated that the activation and polarization of macrophages is regulated by a variety of transcription factors. For example, the transcription factor homeobox A1 is involved in phenotypic transformation of macrophages in atherosclerotic diseases (12). At the same time, the review by Wang et al. in this research topic provides an updated view on the transcriptional regulation mechanism of macrophages in the heart failure stage after acute myocardial infarction and its impact in TCM treatment. In this review, the authors summarize that macrophage phenotypes can be targeted for regulation by inflammatory gene or repair gene transcription. They also discussed other actors in the transcriptional regulatory stage, such as transcriptional cofactors (e.g., Ankyrin repeat domain 1), chromatin modifiers (e.g., zeste homolog 2, histone deacetylase 3), which also affect macrophage regulation of cardiovascular disease. Finally, the authors provide some insights into the mechanism of action and transcriptional regulation of TCM in the treatment of heart failure.

### Transcription regulation between microgravity and cardiovascular disease

The development of aerospace technology means that astronauts who fly in space for a long time now and even in the future may undergo non-negligible changes in body functions and heart organs under the influence of microgravity (13). The research article by Liu et al. provides an updated perspective on changes in the transcriptome and metabolome of rat myocardium under the influence of microgravity. Long-term microgravity can induce myocardial atrophy, causing cardiac insufficiency. The authors used tail suspension to simulate weightlessness in the space environment, and then used RNA sequencing, functional analysis, metabolomics analysis and other experimental methods to reveal that abnormal activation of transcription factors involving Foxo signaling pathway and amino acid metabolism affects the formation of transcriptional regulatory loops, which have the potential to become potential biomarkers and new therapeutic targets for microgravity-induced myocardial injury.

### Novel bioinformatics analysis of transcription regulation in cardiovascular diseases

The research model of multidisciplinary interaction is a prospective and promising form suitable for the development of cardiovascular mechanism research. Using the principles and technologies of bioinformatics to process the genes, transcriptomics, proteomics, drugs, biological networks and other items of various tissue samples of cardiovascular diseases for data mining and analysis, which can provide effective data support for pathological mechanism research, early diagnosis, drug mechanism of action, etc. Existing studies have proved that metabolomics, lipidomics and proteomics comparative analysis of human cardiomyocytes treated with high glucose concentration or empagliflozin treatment have very effectively revealed that empagliflozin treatment improves the phenomenon of high glucose-induced cellular stress (14). Guo et al. verified by linking bioinformatics analysis and molecular biology experiments to verify that transcription factor Tcea3 affects cardiac remodeling by participating in fatty acid oxidation, ATP synthesis, and mitochondrial oxidative stress. Finally, Ping et al. used more detailed bioinformatics analysis techniques to identify ten key pivot genes such as LCP2, PTPRC, RAC2 and CCR2 for cardiac aging, confirming that there is a general negative correlation between age and immunity. It also reveals the importance of detailed bioinformatics analysis techniques such as ESTIMATE algorithm, single-sample gene set enrichment analysis, Pearson correlation screening, weighted gene co-expression network analysis and molecular docking simulation to evaluate the pathogenesis and therapeutic targets of cardiovascular diseases. At the same time, it is further shown that the emergence of emerging technologies such as high-throughput sequencing technology, gene chip (microarray) and genome chromatin analysis technology is conducive to the study of the mechanism of cardiovascular disease.

### Perspectives

In summary, this research topic aims to provide new discoveries and insights on the transcriptional regulation mechanism of cardiovascular diseases (such as myocardial infarction, heart failure, myocardial hypertrophy, etc.), in anticipation of new therapeutic targets or strategies for the treatment of cardiovascular diseases. The above series of research topics also highlights the importance of the linkage between molecular biology experiments and bioinformatics analysis techniques in the development of cardiovascular diseases.
